# Trauma-induced coagulopathy: impact of the early coagulation support protocol on blood product consumption, mortality and costs

**DOI:** 10.1186/s13054-015-0817-9

**Published:** 2015-03-12

**Authors:** Giuseppe Nardi, Vanessa Agostini, Beatrice Rondinelli, Emanuele Russo, Barbara Bastianini, Giovanni Bini, Simona Bulgarelli, Emiliano Cingolani, Alessia Donato, Giorgio Gambale, Giulia Ranaldi

**Affiliations:** Department of Shock and Trauma Center, S Camillo-Forlanini Hospital, Circonvallazione Gianicolense 87, Roma, 00152 Italy; Departement of Clinical Pathology and Transfusion Medicine, Bufalini Hospital, Via Ghirotti 286, Cesena, 45072 Italy; Department of Hematology and Transfusion Medicine, S Camillo-Forlanini Hospital, Circonvallazione Gianicolense 87, Roma, 00152 Italy; Department of Anesthesia and ICU, Trauma Service, Bufalini Hospital, Via Ghirotti 286, Cesena, 45072 Italy; Department of Anesthesia and Intensive Care, University of Siena, Strada delle Scotte 14, Siena, 53100 Italy

## Abstract

**Introduction:**

Hemorrhage is the principal cause of death in the first few hours following severe injury. Coagulopathy is a frequent complication of critical bleeding. A network of Italian trauma centers recently developed a protocol to prevent and treat trauma-induced coagulopathy. A pre-post cohort multicenter study was conducted to assess the impact of the early coagulation support (ECS) protocol on blood products consumption, mortality and treatment costs.

**Methods:**

We prospectively collected data from all severely injured patients (Injury Severity Score (ISS) >15) admitted to two trauma centers in 2013 and compared these findings with the data for 2011. Patients transfused with at least 3 units of packed red blood cells (PRBCs) within 24 hours of an accident were included in the study. In 2011, patients with significant hemorrhaging were treated with early administration of plasma with the aim of achieving a high (≥1:2) plasma-to-PRBC ratio. In 2013, the ECS protocol was the treatment strategy. Outcome data, blood product consumption and treatment costs were compared between the two periods.

**Results:**

The two groups were well matched for demographics, injury severity (ISS: 32.9 in 2011 versus 33.6 in 2013) and clinical and laboratory data on admission. In 2013, a 40% overall reduction in PRBCs was observed, together with a 65% reduction in plasma and a 52% reduction in platelets. Patients in the ECS group received fewer blood products: 6.51 units of PRBCs versus 8.14 units. Plasma transfusions decreased from 8.98 units to 4.21 units (*P* <0.05), and platelets fell from 4.14 units to 2.53 units (*P* <0.05). Mortality in 2013 was 13.5% versus 20% in 2011 (13 versus 26 hospital deaths, respectively) (nonsignificant). When costs for blood components, factors and point-of-care tests were compared, a €76,340 saving in 2013 versus 2011 (23%) was recorded.

**Conclusions:**

The introduction of the ECS protocol in two Italian trauma centers was associated with a marked reduction in blood product consumption, reaching statistical significance for plasma and platelets, and with a non-significant trend toward a reduction in early and 28-day mortality. The overall costs for transfusion and coagulation support (including point-of-care tests) decreased by 23% between 2011 and 2013.

## Introduction

Hemorrhage is the principal cause of death in the first few hours following severe injury. Coagulopathy is a frequent complication of hemorrhage and may occur in up to 25% of patients, even before hospital admission [1]. Recently, a multidisciplinary, pan-European group of experts launched the Stop the Bleeding Campaign [2], a campaign to counteract preventable deaths from uncontrolled bleeding following traumatic injury. The goal of the campaign is to reduce the number of patients who die within 24 hours after hospital admission (due to exsanguination) by a minimum of 20%. This goal was set in response to the updated European guidelines for management of bleeding and coagulopathy [3]. These guidelines recommend that every trauma facility implement an evidence-based treatment algorithm for the bleeding trauma patient and promote the use of treatment algorithms to guide clinical management.

**Figure 1:**
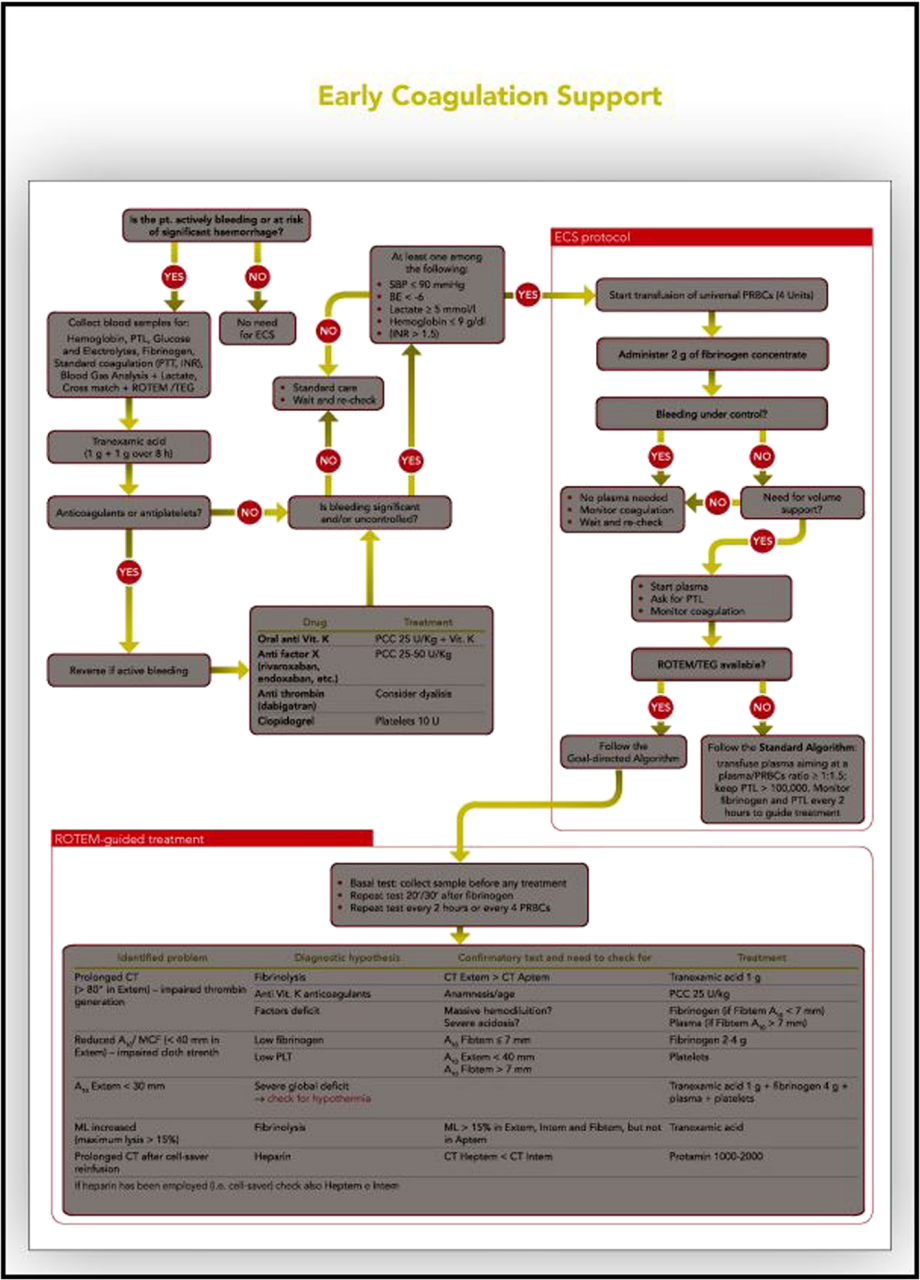
**The early coagulation support (ECS) protocol.**

The Italian Trauma Centers Network (TUN) responded to the recommendations by developing a treatment algorithm (early coagulation support (ECS)). The ECS protocol (Figure 1) has been described in detail elsewhere [4]. It is an integrated part of a comprehensive damage resuscitation strategy. It is also based on fluid restriction and the prohibition of colloids. The ECS strategy has been formally adopted by several Italian trauma centers. To our knowledge, this is the first example in Europe of the adoption of the same step-by-step algorithm for the management of trauma-induced coagulopathy by a large number of trauma centers. The centers that cooperated in the development of the protocol jointly decided to monitor the impact of ECS on consumption of blood products, trauma mortality and morbidity, and financial costs. A multicenter prospective cohort study with retrospective control was therefore planned by the TUN steering committee.

We report the preliminary results of the ongoing study, based on first-year data from the first two trauma centers that implemented ECS.

## Material and methods

Our study was planned as a multicenter prospective cohort study with a retrospective control. According to the original study design, the TUN-associated institutions were required to join the study at the same time. However, the process of adopting the ECS protocol took significant and varying amounts of time from hospital to hospital. On 1 January 2013, only two of the trauma centers were ready to start the study.

We present the results of the first year of use of ECS at the first two sites that adopted the protocol: S Camillo Hospital in Rome and Bufalini Hospital, Cesena, Italy. Both hospitals are high-volume level I trauma centers that serve a population of about 2 million each. Data from the post-ECS adoption period (1 January 2013 to 31 December 2013) and the pre-adoption period (1 January 2011 to 31 December 2011) were compared. In the pre-adoption period, patients were treated according to the 2010 European guidelines [5]. Patients with critical bleeding received plasma as early as possible in a plasma-to-packed red blood cells (PRBCs) ratio aimed at 1:1. Plasma was thawed soon after the patient was admitted, although in a limited number of cases the thawing process could be anticipated based on the pre-hospital team alert. If required, platelets (PTL) were administered with the aim of maintaining the PTL count ≥100,000, as recommended by the guidelines. Coagulation was monitored mainly by means of the traditional laboratory tests (international normalized ratio, prothrombin time, activated partial thromboplastin time, fibrinogen by Clauss method), as point-of-care (POC) tests were not available. The main characteristics of the two different treatment and monitoring strategies are shown in Table 1.

**Table 1:** **Main characteristics of the two treatment strategies** ^**a**^

**Treatment strategies**	**Standard treatment (2011)**	**Early coagulation support (2013)**
Fluid resuscitation	Crystalloids and colloids	Crystalloids only
Fluid treatment strategy	Permissive hypotension, relative fluid restriction (except in TBI patients)	Permissive hypotension, relative fluid restriction (except in TBI patients)
Tranexamic acid	1 g + 1 g 8-hr infusion	1 g + 1 g 8-hr infusion
Initial coagulation support	“Initial” plasma at high level (>1:2) plasma:PRBC ratio	Fibrinogen 2 g
Treatment of uncontrolled bleeding	Plasma:PRBC ratio (>1:2) Keep PTL >100,000	Goal-directed treatment (including plasma:PRBC >1:2 if indicated)
Coagulation monitoring	Traditional: INR, aPTT, PTL, fibrinogen (von Clauss method)	Viscoelastic POC

^a^aPTT, Activated partial thromboplastin time; INR, International normalized ratio; POC, Point of care; PRBC, Packed red blood cells; PTL, Platelets; TBI, Traumatic brain injury.

In 2013, patients were treated according to the ECS algorithm. In both institutions, POC rotational thromboelastometry (ROTEM) was systematically used to monitor coagulation. However, although a blood samples for POC were collected on admission, the initial treatment was based only on clinical signs and on the results of the initial blood gas analysis. Therefore, patients with signs of severe hypoperfusion received the initial coagulation support even before the results of the first (basal) ROTEM test. According to the ECS protocol, patients who met one or more of the following criteria (“clinical” criteria) received a standard dose of fibrinogen concentrate (2 g), together with the first units of universal blood: systolic blood pressure (SBP) <100 mmHg, lactate ≥5 mmol/L, base excess (BE) ≤6 or hemoglobin ≤9 g/dl. POC tests (ROTEM *delta*; Tem International, Munich, Germany) were subsequently used systematically to monitor coagulation and guide the treatment algorithm. Plasma was not administered in the early treatment stage. Permissive hypotension and moderate fluid restriction were recommended until surgical bleeding control was achieved. However, in cases of prolonged bleeding, if an increasing number of PRBCs was required and high volume support was needed, plasma was transfused for volume and coagulation support. In both study periods, patients were resuscitated according to a damage control resuscitation strategy [6] with the aim of limiting the amount of fluids before surgery. However, whereas in 2011 colloids were allowed, the ECS protocol used in 2013 recommended not to use them. During both time periods, the administration of tranexamic acid soon after admission was a mandatory part of treatment [7].

For the purpose of this study, we chose to use for the control group the data of 2011 rather than those of 2012 to allow a 12-month gap between strategies. This ensured that protocols would not overlap and results would not be skewed.

At both hospitals, data for all severely injured patients (Injury Severity Score (ISS) >15) admitted to the intensive care unit (ICU) and those who die before they can be admitted are entered into the trauma center’s database. Trauma patients transfused with at least 3 U of PRBC within 24 hours of a traumatic injury were identified via blood banks’ electronic registries as study candidates. Data from the ICU and emergency room electronic records were matched with the blood bank registries to confirm the blood units transfused within the study’s time spans. For patients transferred from other hospitals, transfusion data before referral and/or during transport were also collected and verified by the blood banks. Information about all patients who met the study criteria were entered into a multicenter database.

### Inclusion criteria

All severely injured patients admitted directly to the trauma centers or transferred by another institution within 6 hours of an accident were candidates for inclusion if they met the following criteria: ISS >15; received at least 3 U of PRBC within the first 24 hours following the accident; either admitted to the ICU or died after hospital admission but before ICU admission, either in the emergency department or in the operating room (OR).

### Exclusion criteria

Patients who had a cardiac arrest prior to admission (or who died en route to the hospital) were excluded. Patients transferred more than 6 hours after an accident or with incomplete medical reports were also excluded.

### Definitions

To allow homogeneous collection of transfusion data, standard definitions of blood components were used. Each unit of PRBC has an average volume of 250 (+10%) ml and a hematocrit of 50% to 60%. The amount of blood obtained through recovery techniques and reinfused was computed considering each 250 ml of blood reinfused equivalent to 1 U of PRBC. Plasma is available either as 200 ml/U of pathogen-inactivated plasma (PlasmaSafe; Kedrion, Barga, Italy) or as single-donor apheresis plasma (600 ml/U). For the purposes of this study, 200 ml of plasma are considered equal to 1 U. PTL are available either as single-donor apheresis or as multiple-donor packs. For the purpose of this study, a single-donor pack is considered equal to six multiple-donor packs. Fibrinogen (Haemocomplettan; CSL-Behring, Marburg, Germany) is available as a 1-g lyophilized, purified human concentrate. Fibrinogen concentrate is not available on the market in Italy, and hospitals are supplied with limited amounts on the basis of authorization from the Italian Medicines Agency at a fixed price of €400/1 g. Industrial plasma costs on average €75/200 ml. There is no established commercial price for PRBC and PTL. However, a reference cost is based on current published figures [8,9]: €186 for 1 U of PRBC and €115 for 1 U of PTL concentrate.

All causes of death were included. Mortality within the first 24 hours was mainly due to hemorrhage. Patients meeting the inclusion criteria who subsequently developed brain death were considered dead after 24 hours if the organ harvesting took place more than 24 hours after the accident in a heart-beating donor.

### Statistical analysis

Distribution of the data was tested with the Kolmogorov–Smirnov test. Data are presented as mean with standard deviation or median with interquartile range, depending on the underlying distribution. Continuous variables were compared using the Mann–Whitney *U* test or Student’s *t*-test, depending on the underlying distribution. For categorical variables, the χ^2^ test was used. A *P*-value <0.05 was considered significant.

The study was approved by the Ethics Committee Lazio 1 as a prospective observational study with a retrospective control. It was determined to waive patients’ consent because in 2013 the ECS protocol was the standard of care in the two institutions, after being formally approved by the directors of both hospitals.

## Results

A substantially equal number of severely injured patients were admitted to the two trauma centers in 2011 and 2013 (435 and 431, respectively). However, 30% fewer patients required transfusion of ≥3 U of PRBCs during the first 24 hours in 2013 (n = 96) than in 2011 (n = 130). Patients in the two periods were well matched in terms of demographics, physiologic parameters on admission, anatomic severity index and laboratory data (Table 2). Seventy percent of the patients in both groups were taken directly from the accident to the trauma center, with an average admission time of 69 minutes (±20 minutes). In 2011, patients received an average of 8.14 U of PRBCs versus 6.51 U in 2013 (nonsignificant), with an overall decrease in blood consumption from 1,048 U to 625 U (−40%) (Table 3). In 2011, 33 patients required massive transfusions (MTs), defined as ≥10 U of PRBC) versus 16 in 2013. The average number of PRBC transfused into the MT patients was 17.8 U in 2011 versus 14.9 U in 2013. The average amount of plasma transfused to the patients in the study groups was reduced from 8.98 U to 4.21 U (*P* <0.05), for an overall 65% reduction from 1,167 U to 405 U. The plasma-to-PRBC ratio decreased in 2013 if compared with 2011 for each amount of PRBC transfused (Table 4). The average amount of PTL transfused was reduced from 4.14 U to 2.53 U (*P* <0.05), an overall 52% reduction in PTL consumption from 538 U to 258 U. In 2013, 134 g of fibrinogen concentrate were used by the two hospitals. Fibrinogen was not used in 2011.

**Table 2:** **Patient characteristics** ^**a**^

				**2011**	**2013**	**Missing**	***P*** **-value**
Patients with ISS >15 and ≥3 U of PRBC				130	96		
	Age, yr	Mean (SD)		48.8 (±19.6)	51.1 (±21.6)	–	0.436
	Sex	n (%)	M	95 (73.1%)	65 (67.7%)	–	0.459
			F	35 (26.9%)	31 (32.3%)		
	Direct admission/referral	n (%)	Direct admission	89 (68.5%)	68 (70.8%)	–	0.770
			Referral	41 (31.5%)	28 (29.2%)		
Clinical data							
	GCS score	Median (IQR)		14 (9)	13 (8.5)	1	0.608
	SBP, mmHg	Mean (SD)		108.5 (±32.0)	104.9 (±29.9)	1	0.482
Anatomical injury score							
	ISS	Median (IQR)		32.5 (16)	33 (18)	–	0.702
		Mean (SD)		32.9 (±11.4)	33.6 (±12.1)		
	AIS head	Median (IQR)		2 (4)	3 (4.5)	–	0.343
		Mean (SD)		2.2 (±2.1)	2.4 (±2.0)		
	AIS face	Median (IQR)		0 (0)	0 (0)	–	0.839
		Mean (SD)		0.51 (±1.0)	0.44 (±0.9)		
	AIS chest	Median (IQR)		3 (4)	3 (3.5)	–	0.777
		Mean (SD)		2.6 (±1.8)	2.5 (±1.8)		
	AIS abdomen	Median (IQR)		2 (3)	0 (3)	–	0.036
		Mean (SD)		1.83 (±1.7)	1.36 (±1.7)		
	AIS pelvis and limbs	Median (IQR)		3 (3)	3 (1)	–	0.138
		Mean (SD)		2.1 (±1.6)	2.5 (±1.6)		
	AIS extremities	Median (IQR)		0 (1)	0 (0)	–	0.05
		Mean (SD)		0.36 (±0.6)	0.19 (±0.6)		
Laboratory data							
	pH	Mean (SD)		7.32 (±0.1)	7.29 (±0.1)	56	0.398
	Lactate mmol/L	Mean (SD)		3.15 (±1.7)	3.18 (±2.7)	52	0.094
	BE	Mean (SD)		−4.47 (±3.5)	−4.96 (±5.0)	63	0.997
	Hb, g/dl	Mean (SD)		10.8 (±2.4)	11.2 (±2.1)	13	0.240
	PTL, μl	Mean (SD)		196.6 (±70.9)	205.3 (±77.4)	53	0.439
	INR	Mean (SD)		1.40 (±0.5)	1.38 (±0.6)	24	0.408
	FIB (von Clauss method), mg/100 ml	Mean (SD)		184 (±92.8)	196.0 (±85.4)	69	0.276

^a^AIS, Anatomical injury score; BE, Base excess; FIB EXTREMITIES, Fibrinogen; GCS, Glasgow Coma Scale; Hb, Hemoglobin; INR, International normalized ratio; IQR, Interquartile range; ISS, Injury Severity Score; PRBC, Packed red blood cells; PTL, Platelets; SBP, Systolic blood pressure; SD, Standard deviation.

**Table 3:** **Impact of introduction of early coagulation support protocol on consumption of blood components** ^**a**^

			**2011**	**2013**	**Missing**	***P*** **-value**
Patients with ISS >15 and ≥3 U of PRBC			130	96		
Blood components transfused within 24 hr						
	PRBC (U)	Mean (SD)	8.09 (6.7)	6.5 (4.8)	–	0.149
		Median (IQR)	5 (6.0)	4 (5.5)		
	PTL (U)	Mean (SD)	4.18 (5.9)	2.68 (4.75)	–	0.046
		Median (IQR)	0 (6)	0 (6)		
	Plasma (U)	Mean (SD)	8.97 (9.47)	4.21 (4.61)	–	<0.001
		Median (IQR)	6 (8)	4 (6)		
Outcome						
	Dead within 24 hr	n (%)	8 (6.15%)	3 (3.12%)	–	0.361
	Hospital mortality	n (%)	26 (20.0%)	13 (13.5%)	–	0.218

^a^IQR, Interquartile range; ISS, Injury Severity Score; PRBC, Packed red blood cells; PTL, Platelets; SD, Standard deviation.

**Table 4:** **Changes in plasma-to-packed red blood cells ratio for each amount of packed red blood cells transfused before and after introduction of early coagulation support** ^**a**^

**Study year**		**3 U**	**4 to 5 U**	**6 to 7 U**	**8 to 10 U**	**>10 U**
2011	Patients, n (%)	32 (25%)	39 (30%)	17 (13%)	10 (7%)	33 (25%)
	Average plasma units	3.4	4.2	5.4	8.3	22.1
	Plasma:PRBC ratio	1:1	1:1	0.8:1	1:1	1.2:1
2013	Patients, n (%)	25 (26%)	33 (34%)	10 (10%)	12 (13%)	16 (17%)
	Average plasma units	1.3	2.4	3.4	6.6	10.6
	Plasma:PRBC ratio	1:2.8	1:2	1:2	0.8:1	0.7:1

^a^PRBC, Packed red blood cells; PTL, Platelets.

In 2011, 26 (20%) of 130 patients died within 28 days following their accidents, and 8 of them died as a result of exsanguination in the first 24 hours. In the 2013 group, 13 (13.5%) of 96 patients died within 28 days, and 3 patients died within the first 24 hours. The difference in mortality between the two periods was not statistically significant. Of the 49 MT patients, 14 died: 10 (30%) of 33 in 2011 and 4 (25%) of 16 in 2013.

The estimated costs of blood products are shown in Table 5. Compared with 2011, 2013 saw an overall 48% reduction from €326,818 to €170,220, for a savings of €156,598. The additional cost of fibrinogen was €53,600. The cost for POC tests was €26,663 in 2013 compared with zero in 2011. However, these data also include the cost of tests performed for all bleeding patients in the OR and ICU, so POC test costs for study patients are overestimated. The average cost for blood components was €2,528.80 for each treated patient in the 2011 group versus €1,492.60 in 2013. Including charges for POC tests and fibrinogen, the average cost per patient in 2013 was €2,322, which is still €206 less than the average cost in 2011.

**Table 5:** **Estimated cost for blood, blood components, factors and point-of-care tests over the two periods (2011 versus 2013)**

		**Estimated cost for 1 U**	**2011**		**2013**	
			**Units (N)**	**Overall**	**Units (N)**	**Overall**
PRBC		€186	1,048	€194,928	625	€116,250
Plasma		€60	1,167	€70,020	405	€24,300
PTL		€115	538	€61,870	258	€29,670
	Overall			€326,818		€170,220
	Balance					−€156,598
Fibrinogen		€400 (1 g)	0	0	134 g	€53,600
POC tests			0	0		€26,663
Overall			0	0		+€80,263
	Balance					−€76,335

^a^POC, Point of care; PRBC, Packed red blood cells; PTL, Platelets.

## Discussion

The timely identification of patients who need aggressive hemostatic resuscitation remains challenging [10,11]. The ECS protocol is activated whenever significant uncontrolled bleeding is associated with one or more of the following criteria: SBP <100 mmHg, BE less than −6, lactate >5 mmol/L and/or hemoglobin <9 g/dl. These criteria were chosen by the TUN panel of experts based on data in the literature and on a retrospective analysis of the TUN trauma registers [4]. Fibrinogen drops early in many patients who sustain a severe trauma, and low fibrinogen levels are associated with higher transfusion requirements and increased mortality [12]. Recently, Schlimp *et al*. [13] demonstrated that levels of fibrinogen <150 mg/dl are detected in as many as 73% of patients with admission hemoglobin <10 g/dl and in 63% of those with a BE less than −6. Moreover, Brohi and colleagues [14] found low fibrinogen in 41% of patients who were hypotensive on admission. Thus, the ECS criteria might well identify patients at high risk of hypofibrinogenemia. According to the ECS strategy, bleeding patients with evidence of severe hypoperfusion should receive a standard dose of 2 g of fibrinogen immediately. The decision to start coagulation support with fibrinogen before plasma infusion differs substantially from the standard clinical practice. In the Activation of Coagulation and Inflammation in Trauma (ACIT) study [15], cryoprecipitate was administered only after the first 6 U of blood and after infusion of the first units of plasma. In the PRospective Observational Multicenter Major Trauma Transfusion (PROMMTT) study, the median time from admission to first cryoprecipitate unit administered was 2.7 hours [16]. In the ECS group, fibrinogen was always administered before plasma was started. A similar strategy is recommended in the Austrian guidelines [17]. However, the Austrian guidelines also recommend the early use of thromboelastometry to detect hypofibrinogenemia and guide treatment from the beginning. This strategy might not always be feasible at many Italian trauma centers, owing to a lack of resources and organizational limitations. Therefore, the ECS strategy supports the initial “blind” administration of fibrinogen to avoid any potentially dangerous delay. In the 2013 group, 50 (52%) of 96 patients met the ECS criteria for an immediate dose of fibrinogen. These data are consistent with the Schöchl *et al*. study [18], in which 52% of patients qualified on the basis of viscoelastic tests and received their first dose of fibrinogen within 1 hour of admission. However, in contrast to the Schöchl *et al*. study the first dose of fibrinogen in our present study was administered to half of these patients, before the results of the basal ROTEM test were available and within 30 minutes of admission. Restoring or increasing fibrinogen levels may improve the clotting process, thus allowing a reduction in the amount of allogeneic blood components transfused. Plasma is currently used as a source of fibrinogen. However, although administration of plasma may stabilize fibrinogen levels and avoid a further decrease, plasma transfusions cannot produce a significant reversal of fibrinogenemia unless very high volumes are infused [19]. The ACIT study confirmed these findings [15].

**Figure 2:**
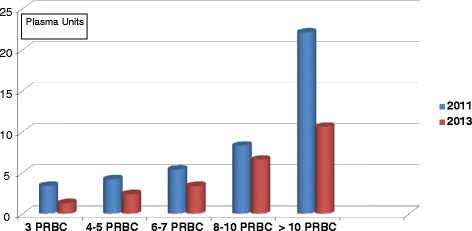
**Changes in the number of plasma units transfused related to the amount of packed red blood cells before and after introduction of early coagulation support.** PRBC, Units of packed red blood cells.

In our study, the number of severe trauma patients (ISS >15) who received ≥3 U of blood was 30% lower in 2013 than in 2011. This difference was independent of any change in the number of major trauma admissions to the two trauma centers and any change in the severity index of the admitted patients. In 2011, immediate administration of plasma to bleeding patients was the cornerstone of coagulation support. The plasma-to-PRBC ratio in 2011 was approximately 1:1 for each amount of PRBC transfused (Table 4). Early and aggressive administration of plasma may result in diluting the blood cells with a decrease in hemoglobin level [20]. This may also reduce PTL marginalization with a potentially negative impact on PTL activation. Moreover, as 7 to 9 g/dl hemoglobin was the transfusion trigger proposed in the 2010 European guidelines [5], the 2011 plasma-based strategy might have triggered a higher amount of blood transfusion, thus increasing the number of patients who received ≥3 U of PRBC. The data on plasma transfusion support this hypothesis. Among the patients transfused with 3 U of blood, the percentage of those who received plasma decreased by more than half in 2013 compared with 2011 (27% versus 56%, respectively), and the average number of units of plasma used in 2013 was less than one-third that used in 2011 (1.2 versus 4.1, respectively). In 2013, the overall number of PRBC units was reduced by 40%, plasma units by 65% and PTL by 52%. Two different reasons are believed to have contributed to the observed decrease in the consumption of blood components: fewer patients who met the study criteria in 2011 compared with 2013 and a reduction in the average amount of PRBCs, plasma and PTL received by each one of the patients in the 2013 group. This difference was statistically significant for plasma and PTL. We observed a consistent reduction of the plasma-to-PRBC ratio for each amount of PRBC transfused (Figure 2). Data in the literature show that early and aggressive plasma transfusion improves the survival of trauma patients with critical bleeding [21-23]. However, availability of pre-thawed plasma is not common in European trauma centers, making early plasma transfusion more a wish than a reality [24]. The average time to start plasma administration in the patients in our 2011 control group was 67 (8-90) minutes. Therefore, the administration of factors as recommended by the ECS protocol might significantly anticipate coagulation support. If fibrinogen concentrate is administered to increase fibrinogen level, there might be less need to transfuse plasma as a source of fibrinogen. Therefore, the lower plasma-to-PRBC ratio we observed in 2013 may be a consequence of the ECS strategy.

In our study, both early mortality and hospital mortality were lower in 2013 than in 2011 (3.12% versus 6.15% for early mortality and 13.5% versus 20.0% for hospital mortality, respectively). However, these are only preliminary data, and the study is not sufficiently powered to demonstrate statistically significant differences. The observed mortality compares favorably with the recent data from the PROMMTT study [25] (21.4%), although the PROMMTT study included patients transfused with as little as 1 U of PRBC and thus the average ISS was lower (26.4%). Patients who undergo MT have a high mortality rate. Mortality rates ranging between 25% [26] and 50% [27-30] have been reported previously, with lower mortality for patients who received a high ratio of plasma to PRBC. MT patients in our study had a mortality rate ranging from 30% in 2011 to 25% in 2013. Patients in both groups received a high ratio of plasma to PRBC, although in 2011 they received twice as many plasma units as in 2013. This greatly reduced the economic costs of patient care, one of the key focal areas of our study. Görlinger *et al*. [31] demonstrated that goal-directed coagulation therapy based on POC monitoring, together with the use of factors, was associated with a sharp reduction in plasma and PRBC transfusions and a €200,000 decrease in the amount spent on blood products per year. Our findings indicate that the cost of blood and blood components was reduced by 48% in 2013 compared with 2011, for an estimated savings of more than €150,000 for the two study hospitals. Even taking into account the additional costs of fibrinogen and POC tests, there was still a savings of more than €76,000. Moreover, in Italy, there is a severe shortage of blood; therefore, saving blood and blood components has relevance independent of the economic impact.

Our study has several limitations. First, it is not a randomized controlled trial. Large, well-conducted studies with pragmatic endpoints are required to improve understanding of the complex interplay between bleeding and coagulopathy, transfusion requirements and mortality. However, these studies are extremely costly, which hinders their feasibility. For this reason, even the most recent recommendations for prevention and treatment of trauma coagulopathy could be based only on the results of observational studies. Our study shows that the introduction of a strategy based on ECS by means of factors administration, together with the use of viscoelastic POC and the ban of the use of colloids, was associated with a reduction in the need for blood and blood components. However, we were not able to define which of these contributed to the achievement of the results for each of the different components of the ECS strategy.

A restrictive fluid resuscitation protocol was used in both periods, although colloids were allowed in 2011 and not in 2013. The 2011 patients received 801 (±545) ml of 130.4 hydroxyethyl starch (HES) (Voluven; Fresenius Kabi, Bad Homburg, Germany) as an average. Gelatins were not used at all. HES may impair coagulation [32,33] and encourage bleeding, thus increasing the need for transfusion. Therefore, the choice to avoid colloids according to the 2013 ECS strategy might have had a relevant role in reducing iatrogenic coagulopathy.

In the ECS group, ROTEM was used to monitor coagulation. When POC is used, the finding of normal viscoelastic curves may support a more conservative approach to plasma transfusion [34]. The systematic use of thromboelastometry POC by the two trauma centers was introduced together with the ECS protocol as an integrated part of the same strategy. Therefore, the possibility to monitor coagulation by means of POC tests is a relevant difference between the 2011 and 2013 strategies. A sharp reduction in patient exposure to allogeneic blood products after the introduction of a thromboelastometry-guided coagulation factor concentrate-based therapy has been reported previously by Schöchl *et al*. [35]. Therefore, on the basis of our results, we cannot state that the early administration of fibrinogen itself, if not associated with the other part of the strategy, is superior to the immediate administration of plasma, which is also recommended in the 2013 European guidelines. Despite the constraints of being an observational study, the results of the present study come from strictly controlled data, thanks to the existing electronic ICU records and blood bank databases. It must be stressed that the data presented here are just the preliminary results. For a better understanding of the impact of the ECS strategy, more data on morbidity (incidence of sepsis, acute respiratory distress syndrome, renal failure) and ICU and hospital lengths of stay are needed. The collection of these data is part of the study project. The analysis is ongoing and will be available within the next few months. The research study continues to involve more institutions, and it is hoped that the increasing numbers of patients included in the study will overcome some of the current limitations.

## Conclusions

TUN recently developed a treatment protocol for bleeding trauma patients called *early coagulation support*. The ECS protocol must be considered as part of a comprehensive damage resuscitation control strategy. The introduction of the ECS protocol in two Italian trauma centers was associated with a marked reduction in blood product consumption, reaching statistical significance for plasma and PTL, and with a non-significant trend toward a reduction in early and 28-day mortality. The overall costs of transfusion and coagulation support (including POC tests) decreased by 23%. These data need to be confirmed with an adequate number of patients. This is the aim of a larger, ongoing multicenter study.

## Key messages

- In every hospital, there is a need for protocols for managing critical bleeding and prevention of coagulopathy.
- The use of the early coagulation support protocol developed by the Italian Trauma Centers Network was associated with a marked reduction in the consumption of blood and blood components, with an associated cost saving.
- There is a strong need for controlled trials to confirm these data.

## Abbreviations

ACIT: Activation of Coagulation and Inflammation in Trauma study
AIS: Anatomical injury score
aPTT: Activated partial thromboplastin time
BE: Base excess
ECS: Early coagulation support
FIB: Fibrinogen
GCS: Glasgow Coma Scale
Hb: Hemoglobin
HES: Hydroxyethyl starch
ICU: Intensive care unit
INR: International normalized ratio
IQR: Interquartile range
ISS: Injury Severity Score
MT: Massive transfusion
OR: Operating room
POC: Point of care
PRBC: Packed red blood cells
PROMMTT: PRospective Observational Multicenter Major Trauma Transfusion study
PT: Prothrombin time
PTL: Platelets
ROTEM: Rotational thromboelastometry
SBP: Systolic blood pressure
SD: Standard deviation
TBI: Traumatic brain injury
TUN: Italian Trauma Centers Network
